# Migration integration policies as social determinants of health for highly educated immigrants in the United States

**DOI:** 10.1186/s12889-023-16254-x

**Published:** 2023-07-14

**Authors:** Mitra Naseh, Yingying Zeng, Abha Rai, Ian Sutherland, Hyunwoo Yoon

**Affiliations:** 1 Brown School, Washington University in St. Louis, 1 Brookings Dr, St. Louis, MO 63130 USA; 2 Center for Immigrant and Refugee Accompaniment School of Social Work, Loyola University Chicago, 1 E Pearson St, #532 Maguire Hall, Chicago, IL 60611 USA; 3 School of Social Work, Portland State University, 1800 SW 6th Ave, Portland, OR 97201 USA; 4 Department of Social Welfare, Kongju National University, 56 Gongjudaehak-ro, Gongju, Chungcheongnam-do South Korea

**Keywords:** Social determinants of health, Migration policies, Highly educated immigrants, Migration, Immigrants, Health

## Abstract

Highly educated immigrants are part of the growing population of immigrants who are impacted by the increasingly hostile migration policies in the U.S. This qualitative study used a phenomenological approach and inductive reasoning to explore the possible impacts of migration integration policies as social determinants of health among this group. Data was collected through 31 semi-structured interviewees with highly educated immigrants who had an intention and interest to stay in the U.S. at the time of the interview. Data were analyzed using reflexive thematic analysis and four main themes emerged: (1) a life overshadowed by silent worries, (2) living through uncertainties and forced decisions as the result of migration integration policies, (3) complexities and challenges of living on a work visa, and (4) shared recommendations by interviewees. Documented narratives as part of this study suggest high rates of stress and anxiety as well as negative mental and physical health outcomes among the participants. Results also suggest high levels of internalized vulnerabilities. Participants shared that migration policies can be enhanced in the U.S. to support highly educated immigrants’ growth by creating a better and more transparent communication system, replacing random review processes for applications with systematic procedures, creating expedited pathways to citizenship based on merit, and granting access to work as a basic human right.

Highly educated immigrants (HEIs), or immigrants with graduate degrees, are part of the growing population of immigrants who are fostering innovation, economic growth, and knowledge sharing in the U.S. (Bernstein et al., 2018 [5]; Hunt & Gauthier-Loiselle, 2010 [13]). They are bringing scarce skills, contributing disproportionately to innovation, and promoting job creation in the U.S. (Morales, 2021 [19]). In 1970, this group accounted for about 7% of the immigrant population in the U.S. (Fry, 2015 [12]). By 2018, this number tripled, and the population of HEIs reached 25% of the total population of immigrants in the country (Batalova & Olsen-Medina, 2020 [4]; Fry, 2015 [12]). Immigrant populations have increased in recent years, and in response migration policies in the U.S. have become increasingly restrictive (Donato & Amuedo-Dorantes, 2020 [11]). Similar to the other groups of immigrants, HEIs are impacted by the increasingly hostile migration policies in the U.S.

Health outcomes for immigrants are most commonly discussed in relation to policies that promote health, assimilation, and integration. However, evidence exists to suggest associations between non-health-directed migration policies and health outcomes among immigrants (Castañeda et al., 2015 [8]; Davis et al., 2006 [10]; Jasso, 2011 [14]). For instance, visa status is increasingly recognized as an integral part of the immigrant experience in the U.S. (Jasso, 2011 [14]; Torres & Young, 2016 [27]). In a quantitative study on immigrants from the Philippines, Morey and colleagues (2020) found evidence that migration policies targeting specific visa categories may have unintended consequences on migrants’ health. Similarly, Tsuchiya and colleagues (2023) found an association between visa types and disparities in depressive symptoms among Filipino migrants. In the context of visa status and health, Jasso (2011) introduced the concept of “visa stress” and “visa depression,” and Torres and Young (2016) presented the impact of legal status stratification on the health outcomes of immigrants from a life-course perspective. Jasso (2011) argues that the complex range of possible legal statuses for immigrants creates specific classes and hierarchies that can simultaneously reinforce and challenge existing social stratification patterns. Torres and Young (2016) suggest that the classes and hierarchies created by the legal status of immigrants can result in health disparities throughout their life course. U.S. migration policies for entry, admission, and deportation modulate immigrant health outcomes, and restrictive migration policies were found to create fear and stress and consequently result in negative mental health outcomes (Davis et al., 2006 [10]; Juárez et al., 2019 [15]; Naseh et al., 2023 [17]). While some work has been done on the impact of migration policies relevant to entering the country and visa status (e.g., deportation or entry visa), scant attention has been given to the specific impacts of migration policies on the health of HEIs in the country (Borjas, 2003 [6]; Juárez et al., 2019 [15]; Miranda et al., 2011 [18]).

The positive effects of educational attainment on immigrant socioeconomic status have been noted in the literature (Raghupathi & Raghupathi, 2020 [22]). However, it has also been shown that immigrants received diminished health returns from improved socioeconomic status when compared to non-migrants (Assari et al., 2020 [2]). A key factor that distinguishes HEIs from other immigrant subgroups is their unique social position as the result of their graduate degrees in relation to restrictions that they experience based on their visa types and immigration statuses. HEIs often face additional pressure to play the role of a model immigrant or follow the “model minority” myth. The model minority myth is the cultural expectation that Asian immigrants and Asian Americans are the ideal racial minority group in the U.S. (e.g. have a perfect family or are perfect employees), especially in contrast with other racial groups (Rai et al., 2022 [23]; Yi & Museus, 2015 [31]). Though ostensibly targeting Asian Americans and Asian immigrants, the model minority myth sets expectations for desirability that extend to all ethnic and racial minority groups in the U.S., including HEIs. For immigrants, the effort or expectation concerning desirability can also be tied to perceived priority for naturalization or suitability for lawful permanent residency. Such misconceptions and expectations can elevate mental health issues, acculturative stressors, and other compounding health factors for immigrants (Sharma et al., 2020 [25]). Acculturation stress refers to the psychological impact of adapting to a new culture, social norms, values, expectations, and ways of life (Thomas, 1995 [26]). HEIs who may feel the need to adapt to the host country’s social norms may experience higher levels of acculturation stress.

Frequently the professional, academic, and social capital that an immigrant had in their country of origin does not receive equivalent recognition in the U.S.; this undervaluation of human capital can create additional barriers to finding a job or maintaining a previously established social position and cause additional stress for HEIs (Bardin & Porten, 1996 [3]; Potocky & Naseh, 2019 [21]). Some of the difficulties of navigating educational and specialized work visas are known (Abdel-Aziz et al., 2020 [1]; Lertora & Croffie, 2020 [16]). HEIs on visas often face stringent work restrictions, which can create additional challenges and stress for them (Abdel-Aziz et al., 2020 [1]; Lertora & Croffie, 2020 [16]). For instance, HEIs who have employment-based visas or employment-sponsored petitions for permanent residency might not be able to change jobs or even accept a promotion for years once their application process has begun (Obinna, 2014). The annual numerical limit for work visas and the country-based quotas of 7% of the total issued permanent residencies for each sending country have resulted in wait times spanning between years and decades for immigrants applying for work visas or permanent residency in the U.S. (Obinna, 2014). Studies have shown possible associations between longer visa or application processing times and depressive symptoms among specific groups of immigrants (Morey et al., 2020 [20]; Tsuchiya et al., 2023 [28]). Restrictions associated with different types of visas for HEIs and the long wait time can also create opportunities for their exploitation and hinder their upward mobility (Naseh et al., 2023 [17]). For instance, many immigrants stay in jobs or educational institutions sponsoring their visas only to keep their visas valid and not because they find their positions fulfilling (Lertora & Croffie, 2020 [16]).

The U.S. migration integration policy is complex and undergoing continuous changes, largely influenced by administrative priorities. At the time of this study’s data collection, the H-1B visa program and Optional Practical Training (OPT) provided HEIs with avenues for temporary stay and work in the U.S., while pathways to citizenship were offered through Employment-Based (EB-1 and EB-2) or Family-Based applications. The H-1B and EB-2 visa (also known as the National Interest Waiver [NIW]), necessitate a sponsor, whereas OPT and EB-1 applications can be self-petitioned. The EB-1 visa category is for individuals with extraordinary ability in the sciences, arts, education, business, or athletics and OPT is designed for graduating students. Applications for H-1B visas sponsored by private sector industries are subject to a lottery system. These applications are accepted once per year during a designated window. All these visa categories come with application fees and intricate application processes. Furthermore, a considerable backlog exists for HEIs from certain countries due to country-based quotas, leading to extended waiting periods.

The systemic and individual-level issues addressed here demonstrate a possible connection between migration policies and the health of HEIs. Our study is focused on migration integration policies and rules and regulations facilitating or hindering HEIs’ access to a path to citizenship. In this study, we explored the role of migration integration policies as social determinants of health among HEIs who had the intention to stay in the U.S. and were planning, were in the process of applying, or recently (less than six months) applied for permanent residency at the time of the interview.

## Methods

This qualitative study used a phenomenological approach and inductive reasoning (Creswell & Poth, 2016 [9]) to document the commonality of lived experiences of 31 HEIs intending to stay in the U.S. in the context of migration policies.

### Sample

After receiving approval from the first author’s university institutional review board, the study was advertised in two online immigration forums in March 2021. Seventy-one HEIs (holding a master’s or doctoral degree from a U.S.-based university) filled out the online demographic survey of the study and filled out an informed consent form agreeing to be contacted for an interview shortly after the advertisement of the study. Fifteen additional participants were recruited during the study (between April 2021 and March 2022) and through referrals from interviewees. Participants who filled out the survey were adult (18 or older) HEIs in the U.S. with valid visas or permanent residencies issued less than six months before their participation in the study. Among the participants who filled out the online demographic survey of the study, 60 electronically consented to have an audio-recorded Zoom interview with our research team. Interviews were conducted by two interviewers in English between April 2021 and April 2022. In the course of conducting 60 interviews, we observed that while immigrants with and without an intention to stay in the U.S. might face similar stressors, these stressors impact HEIs in distinct ways. For this study, we specifically included interviews that clearly expressed an intention or desire to remain in the U.S., resulting in a sample size of 31 participants. We intentionally excluded HEIs without interest or intention to stay in the U.S., enabling us to focus on the effects of migration integration policies, as well as the rules and regulations that either facilitate or hinder HEIs’ pathway to citizenship.

### Data

The online demographic survey of the study collected information on age, gender, country of origin, years in the U.S., education, marital status, annual income, and visa status of the participants. Semi-structured interviews of the study were conducted based on an interview protocol and collected information about the experiences of participants as immigrants, potential stressors in their life, their support system, migration policies, and their recommendations to enhance migration policies to foster the well-being of HEIs in the U.S. Interviews were audio-recorded and transcribed using Zoom. The Zoom-generated transcripts were edited by the research team to ensure accuracy. In the process of editing the transcripts, any identifiable information was redacted. Interviews lasted between 25 and 75 min.

### Analysis

Edited transcripts were analyzed using the reflexive thematic analysis method (Braun & Clarke, 2006 [7]). This process entailed repeated reading and review of the content by the research team, fostering a deeper understanding of the material and facilitating the identification of potential themes. To enhance the rigor of the study and minimize bias, each interview was reviewed and coded by two independent coders. The initial five interviews were open-coded independently, and the generated codes, along with the criteria for code assignment, were discussed to construct a codebook. This codebook was then used to guide the independent coding of all remaining interviews, applying the established criteria for code assignment. Generated codes were merged using a thematic matrix to assign a unique code to similar sections of an interview (Ritchie et al., 2013 [24]). The inter-coder reliability calculated during this merging process was approximately 80%. The consolidated codes facilitated the generation of themes, which were then discussed among the co-authors in a Zoom meeting until a consensus was reached. To further enhance the rigor of the study, peer debriefing and negative case analysis were employed before finalizing the themes.

## Results

The age range of the study participants was between 22 and 40 (Mean = 27.94, SD = 4.08). Around half of the sample self-identified as female (52%, n = 16), and the other half as male (n = 15). The majority (68%, n = 21) of the participants in this study lived in the U.S. between two to five years. Less than one-third of the sample was married (29%, n = 9), and the rest chose the category “single” for their marital status. Participants were from 17 countries including Brazil, Canada, China, Colombia, Ghana, India, Indonesia, Iran, Japan, Netherlands, Nigeria, Pakistan, Russia, Rwanda, South Korea, Sudan, and Venezuela. We had five participants with a Ph.D. degree, and the rest of the sample had a master’s degree at the time of the interview. The reported average annual income by the interviewees varied; around 39% of the sample (n = 12) had an annual income between $100,000 and $200,000, 23% (n = 7) selected the $50,000 and $100,000 range, around 16% (n = 5) had an annual income between $25,000 and $50,000, around 16% (n = 5) had an annual income below $25,000, and 4% (n = 1) had an annual income above $200,000. At the time of the interview, around 77% of the participants (n = 24) were on a student visa (F1) or Optional Practical Training (OPT; OPT is a temporary work permit in the form of an extension of a student visa (F1) after graduation). Around 10% (n = 3) of the sample had work visas (H-1B), around 10% (n = 3) were in the process of getting their permanent residency, and one participant was on a dependent work visa (H-4) at the time of the interviews.

Four main themes emerged from the analysis of the narratives shared by the interviewees in our sample: (1) a life overshadowed by silent worries, (2) living through uncertainties and forced decisions as the result of migration integration policies, (3) complexities and challenges of living on a work visa, and (4) shared recommendations by interviewees. The study was conducted during the COVID-19 pandemic which introduced additional challenges as reported by some interviewees (n = 8). Participants referred to their heightened stress due to prolonged visa processing times, feelings of isolation, job loss, and the implementation of hiring freezes as a result of the pandemic.

### A life overshadowed by silent worries

Worries about visa status and migration policies were present in the narratives of almost all participants in our study. An immigrant from India explained that many aspects of her life depend on her visa status. She said, “There are nights I can’t sleep, and I know it’s just not the visa, it’s a lot of things, because it’s all, like, half of my things, or anybody else’s, people like me, that depends on your visa.” She added that “There is no time to enjoy [life]. I feel like you’re always just thinking about the immigration… It’s, like- every, like- eventually builds up to a place where you feel everything in your life just revolves around your visa.” Similarly, an immigrant from Nigeria stated that her immigration status has been a “major theme” in her life. This interview added that she was so worried about changing her status after graduation that she thought of postponing it; she said, “I was talking with my mentor, I’m like, I’m really concerned, like, I don’t even think I want to graduate yet, … I know once I’m out of school it’s like, okay I’m illegal now.” An immigrant from Russia stated that she feels “powerless” in navigating the immigration system in the U.S. She explained that she is having a hard time understanding why staying in a country that she contributes to and considers home should be this difficult:

*I’m feeling very powerless in front of this monstrous immigration system which makes no sense to me… I speak the language, I respect the laws, I pay my taxes. … I don’t have any ties back to Russia…. I consider United States my home... for the last few years I’ve been in a horrible depression. I’ve been taking antidepressants, I wasn’t able to work, it there were nights where I couldn’t fall asleep for hours- thinking that sometime from now they just going to deport me out of the country.... This whole immigration system is just, it just complete- complete nightmare.*

As part of the interviews, we prompted participants to quantify their stress level while navigating the U.S. immigration system on a scale of zero to ten, with ten as the highest level of stress. All interviewees, except one participant, scored their stress level higher than five. The average self-reported stress score was 7.5 (SD = 1.4). While talking about the stress caused by migration policies, many interviewees referred to negative mental health outcomes they have experienced. For example, a 38-year-old immigrant from India told us that “when I was going through this up and down [related to getting a work visa], I suffered depression…I started relying on Alpax [medication for moderate or severe anxiety] and all those things to get some sleep.” Similarly, a 27-year-old Chinese immigrant told us that she and her husband relied on sleep medication as a result of the anti-Asian sentiment in the country and the stressful process of getting approval for Optional Practical Training (OPT). She said, “There [is] a lot going on outside in the Asian [population], etc., and also the OPT process, I just feel a little bit insecure so it kind of affects myself and my husband’s sleep a little bit, cause he just, had a hard time to sleep, we are relying on melatonin.”

Participants also referred to other health issues such as panic attacks or weight gain as the result of the stressors caused by migration policies. An HEI woman from India said, “Now I am depressed. I’m gaining weight. But my depression doesn’t allow me to go out and take care of myself.” Similarly, a participant from Iran stated, “I’m in [a] medical field, so I know that when I eat emotionally it comes from this kind of stress, so I’d eat; put on a lot of weight during this specific event for the Green Card.” In this context, a participant from the Netherlands referred to her panic attacks during the process of her permanent residency application and said:

*I’ve definitely also experienced panic attacks.... You send it [application for permanent residency] into the big government and the big government decides whether you can stay or not, and whether you can have your life the way that you wish.*

Several participants told us that after applying for any change in their immigration status, their life became consumed by constant worries and stress about the processing time of their application and the decision of the United States Citizenship and Immigration Services (USCIS). A Chinese immigrant waiting for her OPT application to be approved told us that “when I’m working in the daytime, I just keep refreshing that, update, [USCIS] website.” A participant from the Netherlands waiting for her permanent residency application to be processed said, “just like you’re constantly checking your email and your bank account, has the check cashed out yet that I sent to USCIS? Like all this, the constant checking is what gets you, and it is distracting to from my day-to-day work.”

While stress and worry were apparent throughout the recorded interviews, many interviewees stated that they felt they could not share their concerns with others, even their close family members. They told us that most people would not fully understand the nuances of being a HEIs in the U.S. A graduate student from India said that her parents do not understand a lot of her concerns. She said, “sometimes I’ll tell them, ‘oh this bill didn’t get passed and that’s going to make it harder for me to get a Green Card,’ and then they just don’t understand it.” Another participant stated, “my parents are in Brazil and I don’t talk about it [visa issues],” and another HEI from Rwanda shared that “My classmates, right, they are mostly American, so they don’t really understand … I think that the people who understand, who can understand what I’m going through, are just friends that are also immigrants.”

Recorded interviews also illuminated internalized vulnerability in response to reported stress and worries. Some participants blamed themselves or their choices for their worries, and some considered themselves lucky for not experiencing the worst-case scenarios. An architect with a master’s degree told us:

*When I left my country, I knew, like, that would be my life, you know, and I picked my life, no one forced me to pick my stressful life and I picked me, and I am responsible, whatever I am going through, you know...like, I am not even a tree in this country, you know, am I just an immigrant, you know, I don’t have any root, you know… I am a great walker, so I walk every evening, so when I go to the park I see- you know, I see, maybe, you know, what is called, squirrel, or cat, or dog, I just feel like, you know, they are luckier than me because they are not immigrant, you know, they- that’s their country, they don’t have to think about what will happen in tomorrow, you know, they know they are safe, but that’s not me.*

When discussing their coping mechanisms in response to their worries, participants mainly referred to the support of their close family members and their immigrant friends with similar experiences.

### Living through the uncertainties and forced decisions as the result of migration policies

Most participants spoke about uncertainties in their lives and their inability to plan for the future. Constant and random changes in migration policies, long and unclear application processes, and unclear communication with USCIS were among the most cited factors creating uncertainty for the participants.

Regarding changes in migration policies, a student from India said, “[immigration policies] change based on the government, so [when] there’s a new government the policies will change… you can’t like think really long term about staying in the U.S.” An immigrant from Russia referred to a proposed policy by the Trump administration to send international students back if they are not taking in-person classes during the COVID-19 pandemic and told us that she was afraid to be deported. She added that the policy was never implemented, but at the time, she started to think about where to go. Relevant to this topic, an immigrant from Iran shared his frustration about the changes in the national interest waiver (NIW; an application providing a path to permanent residency for an advanced degree or exceptional ability workers) application process during the Trump administration and said:

*I was a top-rated student in Iran, and I could go wherever I want but my dream was high, … I wanted to experience the, the, the educational life in USA. ... So, before 2017, it was, like, for an educated individual like myself…you send your application for NIW, the national interest waiver, that’s the program, and you get your Green Card in mail in six, four to six months. You don’t do anything else. The way he [Trump administration] exactly changed it, when I started this, it took me almost two years and a lot of efforts and unnecessary documentation.*

Participants discussed the long and random review process of specific visas and requests, including OPT. They explained that USCIS reviews OPT applications randomly within a period of time deemed as “normal processing time,” a timeframe that can change and be long as eight months based on the backlog of the applications. Participants added that during the normal processing time, USCIS does not answer follow-up requests and this timeframe can change at any moment. An immigrant from Nigeria called this process “a waiting game,” and that “you have to play the waiting game with the immigration office, you know, because no one is really telling me anything. You’re just waiting for your status to change from whatever it is.” She added that waiting a long period of time for approval of an OPT application is hard, as applicants do not have a right to work during that period. She said, “you have to wait from three to six months [for OPT approval], and what are you going to do for those three to six months, you know? Because you can’t work, because illegal, you know, so it’s very challenging.” Similarly, an immigrant from Sudan said:

*I got, um, I got an [job] offer letter in January itself at the end of the month. I can’t join, I’m waiting for the EAD [Employment Authorization Document] card to be received so I can join my employer; they’ve been nice, they’ve been waiting for me for a month. Last time I contacted them by the beginning of March. And they are still waiting for my application, for my, uh, application to be done and I receive the OPT so I can join them… then like waiting for like four months is going to hurt the finances as well. ... this uncertainty from the, from the, processing [time] and as well as the employer himself, I mean they’re patient, but, yeah, definitely, they won’t be waiting for, forever, right?*

Participants also discussed challenges in applying for work visas (H-1B). They explained that the lottery system of the work visa in the industry has made their lives full of uncertainties. An immigrant on OPT at the time of the interview told us that his company has applied for his work visa this year and the last year, but his application was not selected for review by the USCIS lottery system. He said, “I did have a lot of plans which had to change.” He added that despite all uncertainties in his life, he had to make sure that his work is not impacted so he can keep his job; he told us “We can’t plan ahead, like, we can’t buy any house, or can’t get any loans… It’s kind of a little bit impactful, but we have to make sure that it doesn’t impact our work.” Another participant explained the process of getting a work visa (H-1B) in the industry as part of his interview and said “They’ll [applicants] have to apply for an H-1B and they have three tries, no they have six tries in total, but for students, it’s three [years] while they’re in the U.S… it’s based on a lottery system, it’s not based on a merit system.”

Participants commonly expressed concern about the long process and wait time for residency applications, but this issue was more dramatic for immigrants from certain countries, including India and China, who told us that their wait time could be over a decade. An immigrant from China referred to this process as unfair and explained that “You cannot do anything, basically. You wait for whatever comes to you, like- so you feel, like, powerless. It’s not fair process.” Another participant from India stated that “I feel like I kind of keep putting most of my life on hold based on this [wait time for residency application].” One of the participants said that for some Indians, it is more feasible to wait for their U.S.-born children to turn 18 and apply for their citizenship as the wait time for their own application could be longer than that. She said:

*So, right now, there are certain caps that are imposed on the number of Green Cards given out to each applicant. And I think it’s seven percent per country right now and, at the moment, there are a lot of Indians who are waiting in queue to receive their card and because of these caps there’s only a certain amount of people from each country who get the residency every year, so the queue just keeps growing and then it just takes a lot of time for people born in India or in China to receive their Green Cards and oftentimes I also hear from my friends that our best bet at getting a Green Card is just by having like having kids here and then applying through them once they turn 18 which is this really morbid, but yeah, it just, it takes a long time.*

Uncertainties and restrictive migration policies were linked to an array of forced decisions in the narratives of the interviewees. For instance, a participant talked about getting an additional degree for a second chance in the U.S. work visa lottery. An immigrant from India told us:

*[In my] three years of OPT, they [my employers] applied for my H-1B [work visa] every year and I did not get it. My lottery was not lucky enough. And, you know, the Green Card is pretty messed up for Indians .... So, I basically had to go and get another Master’s degree from [name of the university is redacted] to be able to stay back in the U.S. The first degree was in electrical engineering and I had my second Master’s in engineering and technology management. And, now, on my fourth attempt I got my H-1B and this was in the midst of the pandemic.... morning I was at work, evening I would attend classes twice a week, and then the weekend could go- would be spent in solving assignments, and sometimes work would be busy that I needed to do some work tasks on the weekend. So, I needed to be at it, I didn’t have the luxury of falling sick, had I had COVID.*

Another interviewee from India shared her complex journey and series of forced choices to be able to work as an HEI immigrant in the U.S.:

*I tried to get a job on H-4 [visa for dependents of a noncitizen worker] … starting from square one. But then it didn’t work out. I did not have the H-4 EAD [Employee Authorization Document] at that time because my husband did not have the I-140 petition [petition for a noncitizen worker to become a permanent resident in the U.S.] approved … I was, like I need to do something… I went back to the college ... I was able to do that. Then, the problem was, I needed to do an internship. Now, I cannot do an internship on H-4 [without a right to work]. They don’t allow you to do an internship on H-4. S*o, my *university suggested me to move to an F1[student visa]… so I stayed here; waited, I think, almost a year, nine to 10 months it was, and then when I got my …F1 approval … I was so happy, but it was summer break, so it was almost a year before I could get into that position and get the internship… I had to, you know, extend my studies for at least one semester.*

Participants also told us about years of family separations due to restrictions of their single-entry visas or perceived risks associated with re-entering the country with multiple-entry visas. One of the participants who had a single-entry visa said, “My mother-in-law passed away last year… my husband has two brothers, but due to all these visa issues, none of them could go.” She added, “So, it’s not only us who has to bear the brunt of … these visa issues, it’s also our families back home and here.” An interviewee from the Netherlands explained that she cannot leave her current job and join her husband in another city out of fear of delays in getting work authorization for the new job. She said:

*My husband just recently took a job in a city 10 hours from here so he’s going to be up north, where I’m going to be in [name of the state was redacted] and we had to make the decision for me to stay, just because I can’t count on USCIS giving me a new EAD [Employment Authorization Document] by the time that we’d have to move… so my best bet is to stay where I am and hope that my current employer will re-hire me.*

### Complexities and challenges of living on a work visa

Interviewees on a work visa, those on work visas in the past, and participants in the process of applying for a work visa talked about the complexities and challenges associated with this specific type of visa. Regarding the H-1B work visa, most of the interviewees indicated feelings of powerlessness and anxiety in going through the random and lottery-based application process. An interviewee from India shared her experience applying for an H-1B visa and said:

*The last year was traumatic because, you know, it was kind of a lottery-based visa system… so I did not know my visa will be picked up or not. So, the whole year… it was kind of every night I had to take sleeping pills… because I can’t control my chaos.*

An interviewee from Indonesia on a student visa indicated that she might go to Canada if her application would not be selected in the work visa lottery system because she only had one year of OPT as her legal status to stay in the U.S. The U.S. immigration authorities grant three years of OPT to STEM graduates, but only one year of OPT to non-STEM major students. H-1B applications are often accepted once a year during a certain period marked as the application window. For non-STEM students, this would be their only chance to apply. The interviewee from Indonesia said:

*Yeah, it kind of takes a miracle, you know, because I only have, like, one year OPT and then I don’t know …, you know, it will be thrown in a lottery anyway, but, yeah, that’s the hope and- Yeah, if that doesn’t work, well maybe I would get a- Yeah, I don’t know, I might go to Canada*.

Some participants told us that what makes the lottery system of the H-1B visa worse are the loopholes in the system. They explained that applicants can ask multiple employers to submit applications for them to maximize their chances, but this can hinder the chances of other applicants. An interviewee from Nigeria explained:

*I saw somebody on the internet who sent in twelve applications, and seven of those got picked under the same name, and it’s just ridiculous, you know, because he only needs one to go forward, and then there’s six other slots that have been filled up which could have been taken up by people who weren’t picked, and some people, unfortunately, this was their last time to try for the work visa, and now they are left in the dark as to what happens to them next. So, it’s kind of ironic that a country like the US still hasn’t figured out how to work around that gaping loophole, but that’s something that definitely needs a lot of attention.*

People working in certain organizations, such as in universities and non-governmental organizations, do not need to go through the lottery system to get an H-1B visa, but delays in processing requests can still cause great anxiety for them. An interviewee from Ghana said, “I believe there was technically one day that I had no status. I think my OPT had, like, had just expired before the H-1B notification was given.”

Work visas in the U.S. are often sponsored by employers, meaning that immigrants on these visas can legally stay in the U.S. as long as their employers are willing to keep their visas valid. This system creates more room for abuse of power by employers. In this context, a Korean interviewee said:

*I think because my employer’s processing my visa, I have to be with them for two more years. So, even though I want to move my job I have to stay with this job with unfair wage and- Like, if they don’t give me any raise. So yeah, that’s- so- that’s part, I think... it’s also related to my job, but I want to do other, like, job, but I can’t do it because my visa restriction.*

Because HEIs’ ability to obtain work visas largely depends on employers’ willingness to sponsor them, many immigrants encounter subjective and objective discrimination in the labor market. An interviewee from Rwanda shared her job search journey and explained:

*When you’re applying you have- there’s the option oh, are you going to require sponsorship in the future? And the more you click yes, the more they’re not gonna call you back so it’s definitely stressful … if you don’t know and also applying knowing that you might get rejected just because you have- you require sponsorship in the future.*

Interviewees told us that many employers covertly reject immigrants, and some publicly announce that they do not hire immigrants to avoid challenges associated with work visa sponsorship. An interviewee from Russia referred to work visa sponsorship as a structural barrier and explained:

*I also felt a lot of problems applying for jobs. A lot of them put that right away that we do not sponsor work visas, neither now, nor in the future. So that automatically disqualifies me from a lot of jobs. I honestly do see a lot of those notes that, if you’re an international person, do not even apply to us- neither now, nor in the future, we cannot sponsor your visa. So, I don’t know where to find that unicorn position that can actually satisfy my level of knowledge, pay normal money, and actually sponsor me.*

### Shared recommendations by interviewees

As part of the interviews, participants told us about ways to improve the migration system and proposed policies that would foster growth and mental health among this group. Recommendations discussed by interviewees were grouped into four main categories: (1) better and more transparent communication, (2) systematic review processes for applications, (3) access to work as a basic human right, and (4) expedited pathways to citizenship based on merit.

#### Better and more transparent communication.

An immigrant from Sudan said that he avoids contacting USCIS since a machine will respond and will “copy and paste what’s in the website.” He added “for us, being able to talk with a human agent from the USCIS would relieve a lot of stress. So we can know what’s actually happening, you know, with our application.” In regards to the lack of communication, an immigrant from the Netherlands said, “not having a person to reply, or anything; that specifically was challenging in the process.” Many interviewees said that in the absence of clear information and communication by USCIS, they have used unofficial platforms to get information about their application status. An immigrant from Venezuela said:

*Reddit forums, online trackers, kind of like, give you a really good overview about the process ... I feel like these are amazing resources that people have put together and they should be better for like all the other applications…. And I think that they [USCIS] could do so much better, but I feel like they don’t want to. Like, they want to keep the process completely isolated from… the applicants and… it’s frustrating… The system that immigrants created themselves; a[n] OPT tracker, USCIS tracker, OPT timeline, all these things, you know, the public forums - I think those are the ones that actually help you out. I even myself, you know, create my own, you know, tracking script to, like, just pull information from UCIS and kind of… have a more holistic view of what’s going on.*

#### Systematic review processes for applications.

Interviewees referred to the lottery system in the USCIS review process and the wide and constantly changing “normal processing time” and recommended more transparent and systematic review processes. An immigrant from Sudan said that having a structured process for the application review by USCIS can help him with “managing finances, managing time, and having, planning ahead.” A participant from China referred to the lottery system in the review process of work visa applications and called it “ridiculous;” he asked, “why can’t they just do first come first serve thing?” Similarly, a participant from Venezuela who was in the process of applying for OPT said, “I don’t really [think] that needs to be a lottery, it should be first come, first serve. If you apply early, you get a spot … it makes no sense; why it is not simply just first come, first serve and that’s it?” He further talked about the wide USCIS processing timeframes and said:

*I just feel like they put that timeline because they are just trying to cover their bases. And … every time that you ask, hey, how’s my case? It’s still a normal processing time, and it’s like, okay, I mean, that’s true but, like what is this? A normal processing time? I think it’s just biased, so you can never complain about it.*

#### Access to work as a basic human right.

Many interviewees referred to their restricted access to work and asked for more supportive policies that would provide a right to work for HEIs. An immigrant from India who was on a work visa said, “we’re just asking for basic human rights to work.” He added that “I’ve paid $200,000 in U.S. taxes, I’ve contributed to the economy,” and said the U.S. needs a “more permanent work authorization” for immigrants on work visas. A participant on an H-4, a visa with no right to work for dependents of immigrants on a work visa, told us:

*Well, there are many western countries like UK and Canada, who allow dependents to work. There is no restriction which separates the prime visa holder and the dependent in terms of working. And it starts from day one; they get the visa. I would say first thing I would want to change is take that curse away from H-4 EAD or L-2 or any other dependent visa.*

#### Expedited pathways to citizenship based on merit.

Among the other recommendations by interviewees to make more supportive migration policies was the creation of a merit-based point system to prioritize HEIs. A participant from India said, “I’m all for a merit-based immigration system, at least for highly educated immigrants.” Another shared recommendation by interviewees was improving procedures in reviewing permanent residency applications of Chinese and Indians. A participant from India recommended “having a point-based system” and several other interviewees from India recommended removing the “country-based quota” for permanent residency applications.

## Discussion

Findings suggest that migration policies can be conceptualized as social determinants of health for immigrants and restrictive migration policies are associated with negative health outcomes among HEIs. Interviewees repeatedly reported stress, anxiety, uncertainties, and forced decisions in their interactions with migration policies in the U.S. Some reported sleep disturbance, depression, and weight gain as health outcomes associated with restrictive migration policies. Interviewees also discussed the impacts of restrictive migration policies on the other aspects of their lives, including their careers and ability to plan for the future. These findings are in line with Jasso’s (2011) work on “visa stress” and “visa depression,” and Torres and Young’s (2016) discussion about the relationships between legal status and health disparities.

The findings of our study call for the rethinking of vulnerabilities among HEIs, a group that is often considered a privileged group in terms of income or educational background. Vulnerability in the general term refers to susceptibility or exposure to harm. In our study, the susceptibility to harm among HEIs was mainly concerning psychological distress and was caused by migration integration policies. Restrictive migration policies can impact HEIs’ mental health and overall well-being despite their relatively high socioeconomic status (SES). Exploring vulnerabilities only through the lens of SES (e.g., income or poverty) can mislabel HEIs and similar groups impacted by different structural barriers, including restrictive migration policies.

Restrictive migration policies can put HEIs at risk of experiencing deprivations and hardship in different aspects of life. For instance, international students on a student visa (F1) in the U.S. should be enrolled as full-time students to keep their visas valid. Meanwhile, these international students with F1 visas do not have a right to work, except part-time on campus (USCIS, 2020 [29]). Most international students rely on tuition waivers, scholarships, and fellowships to afford a living in the U.S. On a case-by-case basis and when students are in severe economic hardship, they might be able to receive permission to work off campus (USCIS, 2020 [29]). After finishing their first year of education in the U.S., international students can receive permission to work off-campus in fields related to their education through Curricular Practical Training (CPT), OPT, and extended OPT for STEM (USCIS, 2020 [29]). International students can apply for permission to work after graduation through OPT and extended OPT for STEM as well. Permission to work through OPT is often used after graduation and is for a maximum of 12 months. This period can be extended for STEM major students for up to 24 additional months. If a student uses CPT to work while studying, the time on CPT will be deducted from the allowable time on OPT. Compared to permission to work through CPT and OPT, securing a work visa for HEI could be a much more complex process. For work visas (H-1B), HEIs should find employers who would be willing to sponsor their application, pay for it, and go through its random and long review process (USCIS, 2022 [30]). U.S. has an annual numerical limit (65,000 plus 20,000 additional visas for applications with graduate degrees) for H-1B visas (USCIS, 2022 [30]). The complex process of finding a sponsor, the associated fees with the work visa application, and the lottery system of reviewing applications through the private sector industries greatly increase HEIs’ mental burden when seeking jobs in the U.S.

In the era of globalization, human rights should guide migration policies for global citizens. Through analyzing these interviews, we presented multiple cases in which migration policies have neglected human rights and subjected HEIs to institutional, social, and cultural discrimination and exclusion in the U.S. Besides being restrictive, many of the existing migration policies in the U.S. are targeting specific groups of immigrants or have different implications for them. For example, international students from Iran often receive single-entry student visas that can cause years of family separation, the same visa is often multiple-entry for international students from other countries. Or, the permanent residency application process can take over a decade for HEIs from India or China, while this process is often between a year or two for other groups of HEIs. Previous studies have shown associations between visa processing time and negative health outcomes such as depression (Morey et al., 2020 [20]; Tsuchiya et al., 2023 [28]). The role of migration policies is critical in emphasizing the universality of human rights necessitated by a multicultural society. Nevertheless, existing immigration policies in the U.S. have been unsuccessful in protecting the human rights of migrants, including HEIs according to the findings of this study.

### Limitations and strengths

The results of the study are not generalizable based on the qualitative nature of the study. Qualitative studies are inherently subjective and based on the conclusion of the research team (Creswell & Poth, 2016 [9]). Moreover, qualitative studies’ small sample size often makes the results context-specific. Although a limitation, our goal for this study was to provide a more in-depth understanding of the impacts of restrictive migration policies and the qualitative nature of the study ensured the fulfillment of this goal. The phenomenological approach of the study enables us to capture the complexities of the HEIs’ experiences. Participants were recruited through online platforms and referrals from interviewees, which could result in selected bias and homogeneity. We had sample diversity, and it is one of the strengths of our study, but it is likely that HEIs who respond to online recruitment differ from those who did not. In this study, we only report on the shared life experiences of 31 immigrants from 17 countries and did not report or emphasize the impacts of specific policies targeting HEIs from certain countries. For instance, we briefly mentioned the unimaginably long waiting time for permanent residency applications submitted by Indians and Chinese. Future studies may consider focusing on the experiences of immigrants from certain countries and/or using mixed methods or triangulation of different data sources to allow generalizability as well as an in-depth perspective about the experience of HEIs.

Our study is unique as it focuses on an under-studied population of HEIs and explores the intersection of migration policies and health. Moreover, our paper challenges the conventional assumptions about HEIs as a group facing fewer challenges in life by highlighting how restrictive migration policies can significantly impact their mental health and overall well-being.

### Conclusions and recommendations

This study aimed to explore the impact of migration integration policies as social determinants of health among HEIs. Based on semi-structured interviews with 31 HEIs, we concluded that restrictive migration policies are associated with negative health outcomes among HEIs. Interviewed HEIs told us that their lives are overshadowed by silent worries. They disclosed that they are living through uncertainties and forced decisions as a result of migration integration policies and shared the complexities and challenges of living on a work visa. Interviewees of this study provided concrete recommendations about improvements needed in the migration policies and asked for (1) enhanced and transparent communication during the visa application process, (2) the establishment of systematic review procedures for applications as opposed to random reviews and lottery-based methodologies, granting all HEIs the fundamental human right work, and expedited pathways to citizenship based on individual merit.

Many interviewees believed that providing HEIs intending to stay in the U.S. with the option to secure employment or a path to citizenship based on their skills rather than chance, visa type, and major can improve their quality of life. We had participants from India who strongly believed that removing country-based quotas would make the migration integration journey more just and unbiased in the U.S. Given the substantial intellectual and financial contributions of HEIs to the community, it is crucial for them to feel truly welcomed in the U.S.

As discussed by the participants and reported in the previous studies, U.S. migration integration policies are showing limitations in several ways and are associated with negative health outcomes (Morey et al., 2020 [20]; Tsuchiya et al., 2023 [28]). While restrictive migration policies can result in further health inequalities in the U.S., more inclusive policies can foster the growth and contributions of HEIs. Many of the limitations in U.S. migration policies can be resolved with the participation of immigrants in decision-making. The Universal Declaration of Human Rights can be used as a guideline to design more inclusive and human rights-friendly migration policies in the U.S. Additionally, promoting cultural competence among politicians who enact immigration laws and public officials who practice them is also important. Recognizing the experiences of different groups of immigrants and sharing knowledge related to multiculturalism are steps toward promoting social justice and inclusion in the U.S. Further studies are needed to provide an in-depth understanding of the history, traditions, value systems, worldviews, family systems, and artistic expressions of different groups of immigrants in relation to migration policies. Through such information, policies can be designed to embody the realization of human rights for all.

## Data Availability

Anonymized transcripts of the interviews for this study are available on request from the corresponding author.
